# Comparison of the prognostic value of AJCC cancer staging system 7th and 8th editions for differentiated thyroid cancer

**DOI:** 10.1186/s12902-022-01054-y

**Published:** 2022-06-01

**Authors:** Y. J. Morosán Allo, L. Bosio, A. Morejón, C. Parisi, M. C. Faingold, V. Ilera, A. Gauna, G. Brenta

**Affiliations:** 1 Endocrinology Division, Cesar Milstein Hospital, CABA, Buenos Aires, Argentina; 2 Endocrinology Division Ramos Mejía Hospital, CABA, Buenos Aires, Argentina

## Abstract

**Background:**

In the last American Joint Committee on Cancer/Tumor, Node, Metastasis (AJCC/TNM) 8th edition (TNM8), several changes were introduced to this risk stratification system to improve the prognosis of differentiated thyroid cancer (DTC).

**Aim:**

To validate the impact of TNM8 vs. TNM 7th edition (TNM7) in DTC in terms of predictive value in two hospitals from Buenos Aires, Argentina.

**Methods:**

Retrospective study of DTC patients from two institutions. Reclassification from TNM7 to TNM8, disease-specific survival (DSS), and final clinical outcomes at the end of follow-up (recurrent/persistent structural disease) (median 5 years) were analyzed. The proportion of variation explained (PVE) was used to compare the predictive capability of DSS of both classification systems.

**Results:**

Reclassification of 245 patients, aged (mean ± SD) 55 ± 15.36 years, 91% women, to TNM8 from TNM7 showed: 82% vs 57% stage I (SI), 10% vs 8.5% SII, 5% vs 22% SIII, 3% vs 12% SIV (*p* < 0.01). Forty percent of the population was downstaged with TNM8. Ten-year DSS rates for SI, SII, SIII and SIV in TNM7 were 100, 100, 100 and 74%, respectively and in TNM8: 97.6, 100, 100 and 37.5%, respectively. Out of 4 disease-specific deaths in SIV TNM7, one was subclassified to SI TNM8, corresponding to a 53-year-old patient with structural persistence. PVE for TNM8 (29%) was more than twice that of TNM7 (13%).

**Conclusion:**

In this Argentinian DTC patients sample, it was confirmed that the new TNM8 classification is more accurate in predicting survival attributable to cancer than its previous version.

## Background

In October 2016, the American Joint Committee on Cancer published the 8th edition of the American Joint Committee on Cancer / Tumor, Node, Metastasis (AJCC / TNM) classification of differentiated thyroid cancer (DTC), which replaced the 7th edition used worldwide since 2009 [1, 2].

Several modifications have been incorporated; minimal/microscopic extrathyroidal extension (mETE) was removed from the T3 category, divided into two subcategories. The age cut-off was modified from 45 to 55 years [3–7]. In particular, at Memorial Sloan Kettering Cancer Center (MSKCC), Nixon et al. [5] showed for the first time that the 55-year cutoff point improved the prediction of disease-specific survival in high-risk patients.

In brief, the main modifications introduced in AJCC / TNM 8th edition include the new 55 ys old cut-off age and, in particular, differences in staging definitions for older patients, which can be summarized as follows: DTC ≤ 4 cm that is confined to the thyroid is considered SI; the presence of neck lymph node metastases or gross extrathyroidal extension involving only the overlying strap muscles are considered SII; gross extrathyroidal extension into significant structures in the neck without distant metastases is considered SIII and extensive gross extrathyroidal extension defined as T4b disease or distant metastases at diagnosis are considered SIV [1].

Although the new AJCC / TNM 8th edition is deemed more accurate regarding mortality prediction, its diagnostic value in different ethnic populations is still evaluated. Several studies in Europe, Asia, and North America [3–5, 8–13] have been made to describe it, but in Latin America, local evidence is scarce. Therefore, this study aimed to validate the impact of the new AJCC / TNM 8th edition classification in an Argentinian population in terms of mortality prognosis.

A secondary objective was to analyze within each TNM7 and TNM8 stages, the distribution of the 2015 ATA recurrence risk categories and clinical status at the end of follow-up, according to the 2015 ATA guidelines recommendations.

## Methods

We conducted a study in a hospital for older adults (Dr. César Milstein Hospital) and in a general hospital (Ramos Mejía Hospital), located in a metropolitan area of Argentina. Both the Dr. César Milstein Hospital Ethical Committee and the Ramos Mejia Hospital Ethical Committee gave their approval for the protocol of this retrospective study. All methods were carried out in accordance with relevant guidelines and regulations. Informed consent was obtained from all subjects and/or their legal guardian(s).

We retrospectively included patients referred to surgery for DTC at each institution ≥18 years of age, between 6/2008 and 6/2017, as well as those referred from other centers for follow-up of DTC with enough information to be classified according to AJCC / TNM. All patients had received surgical treatment with or without radioiodine ablation following 2015 ATA guidelines and had a follow-up of at least 12 months. Anaplastic and medullary carcinomas and those patients who did not have complete information to be evaluated over time were excluded. All patients or their legal guardian(s) gave their informed consent to the use of data for this study.

Demographic variables, including age at diagnosis, sex, and year of diagnosis, were collected. Disease characteristics included were: histological subtype, tumor size, lymph node and distant metastases, minimal and gross extrathyroid extension, surgical extension, and radioiodine ablative doses. AJCC / TNM stage and 2015 ATA recurrence risk stratification were obtained, and clinical status at the end of follow-up and disease-specific mortality were recorded.

Disease-specific mortality (DSM) was defined as occurring in patients with extensive or rapidly progressive disease without any other apparent cause of death.

Disease-specific survival (DSS) was defined as the period from diagnosis until death or until the last day of follow-up or the end of the study in June 2017.

Clinical status at the end of follow-up was classified as non-evidence of disease (NED), undetermined response, and structural or biochemical persistence according to the 2015 ATA guidelines recommendations [2]. For this study, disease recurrence/persistence included both biochemical and structural recurrence/persistence.

Based on the information gathered, AJCC / TNM 7th (TNM7) and 8th (TNM8) were calculated, and the number of reclassified patients was determined and disease-specific mortality in the different stages.

Recurrence/structural persistence of the disease was also compared between the different stages of the two editions, TNM8 and TNM7, of AJCC / TNM.

The effects of applying TNM8 to patients between 45 and 55 years of age were further analyzed regarding disease outcomes.

### Statistical analysis

Data were presented as mean and standard deviation or median and range, as appropriate for each variable. Chi-squared test or Fisher’s test was used to compare dichotomous variables; *p* < 0.05 was considered significant. Sensitivity, specificity, and likelihood ratio were calculated to predict Stage IV mortality from TNM8 and TNM7. Kaplan Meier survival curves were performed in both stratification systems. The log-rank test was used to determine significance. The impact of both TNM8 and TNM7 classifications on the prediction of DSM was determined by the Cox Proportional Hazard. The proportion of variance explained (PVE) expressed as R Squared (%) was obtained by ANOVA analysis to compare the relative validity of each TNM classification. GraphPad Prism version 5.01 and SPSS version 25.0 (IBM, USA) were used for all statistical calculations.

## Results

### Patients

This retrospective study included 245 patients compatible with the inclusion criteria from two healthcare centers in the metropolitan area of Buenos Aires, Argentina. The baseline and clinical characteristics of the DTC patients are listed in Table 1. Most of the patients, 52% (*n* = 126) were > 55 years old and 91% (*n* = 223) were women. Ninety one percent (*n* = 224) were papillary carcinomas, 7.8% (*n* = 19) follicular carcinomas, 1.2% (*n* = 2) Hürthle cell carcinomas. The median follow-up was 60 months, and during follow-up, four patients died due to DTC.

**Table 1:** Baseline clinical and pathological characteristics of patients with DTC

		Mean ± SD or Number (%)
Age		64 ± 11.3
	< 45	64 (26)
	45–55	55 (22)
	≥55	126 (52)
Sex	Female	223 (91)
	Male	22 (9)
Pathology	PTC	224 (91)
	FTC	19 (7.8)
	Hurthle	2 (1.2)
Tumor size (cm)	≤1	65 (26)
	1--2	76 (31)
	≥2--4	79 (32)
	≥4	22 (9)
Microscopic extrathyroidal extension		56 (23)
Gross extrathyroidal extension		19 (8)
T3b		3 (16)
T4a		16 (84)
Cervical LN metastases		38 (15)
Distant metastases	Total number	6 (3)
	lung	4 (66)
	bone	2 (34)
Initial surgical extent	Lobectomy	8 (6)
	Total thyroidectomy	235 (96)
	Biopsy	2 (2)
RAI treatment		171 (70)

Abbreviations: *DTC* differentiated thyroid cancer, *PTC* papillary thyroid cancer, *FTC* follicular thyroid cancer, *LN* lymph nodes, *RAI* radioactive iodine, *SD* standard deviation

Tumor size was ≤1 cm in 26% (*n* = 65), between 1 and 2 cm in 31% (*n* = 76), between ≥2-4 cm in 32% (*n* = 79), and ≥ 4 cm in 9% (*n* = 22); microscopic ETE was present in 23% (*n* = 56); gross ETE in 8% (*n* = 19); cervical lymph node (LN) metastases in 15% (*n* = 38); distant metastases in 2.4% (*n* = 6, 4 in the lung and 2 in the bone).

Total thyroidectomy was performed in 96% (*n* = 235) of patients, and 70% (*n* = 171) received radioiodine (RAI) ablation.

### Comparison of the number of patients in each stage according to TNM7 and TNM8

Based on TNM7, 139 patients (57%) were assigned to stage I (SI), 21 patients (8.5%) to stage II (SII), 54 patients (22%) to stage III (SIII), and 31 patients (12%) to stage IV (SIV). When we applied TNM8 and compared TNM7 vs. TNM8, it was observed that 40% of the whole population was downstaged: in stage II, 95% of the patients were downstaged to SI, and 5% remained in SII. From SIII, 65% of patients were downstaged to SI and 35% to SII; from SIV, 26% were downstaged to SI, 13% to SII, 39% to SIII, and only 22% remained in SIV. Table 2 shows the number of patients according to each stage of AJCC / TNM after reclassification.

**Table 2:** Number of patients per TNM stage according to TNM7 and TNM8

		TNM8			
		I (*n* = 202)	II (*n* = 24)	III (*n* = 12)	IV (*n* = 7)
	I (*n* = 139)	139	0	0	0
TNM7	II (*n* = 21)	20	1	0	0
	III (*n* = 54)	35	19	0	0
	IV (*n* = 31)	8	4	12	7

Abbreviations: *TNM* tumor-node-metastases, *TNM7* 7th edition of TNM staging system, *TNM8* 8th edition of TNM staging system

**Fig. 1:**
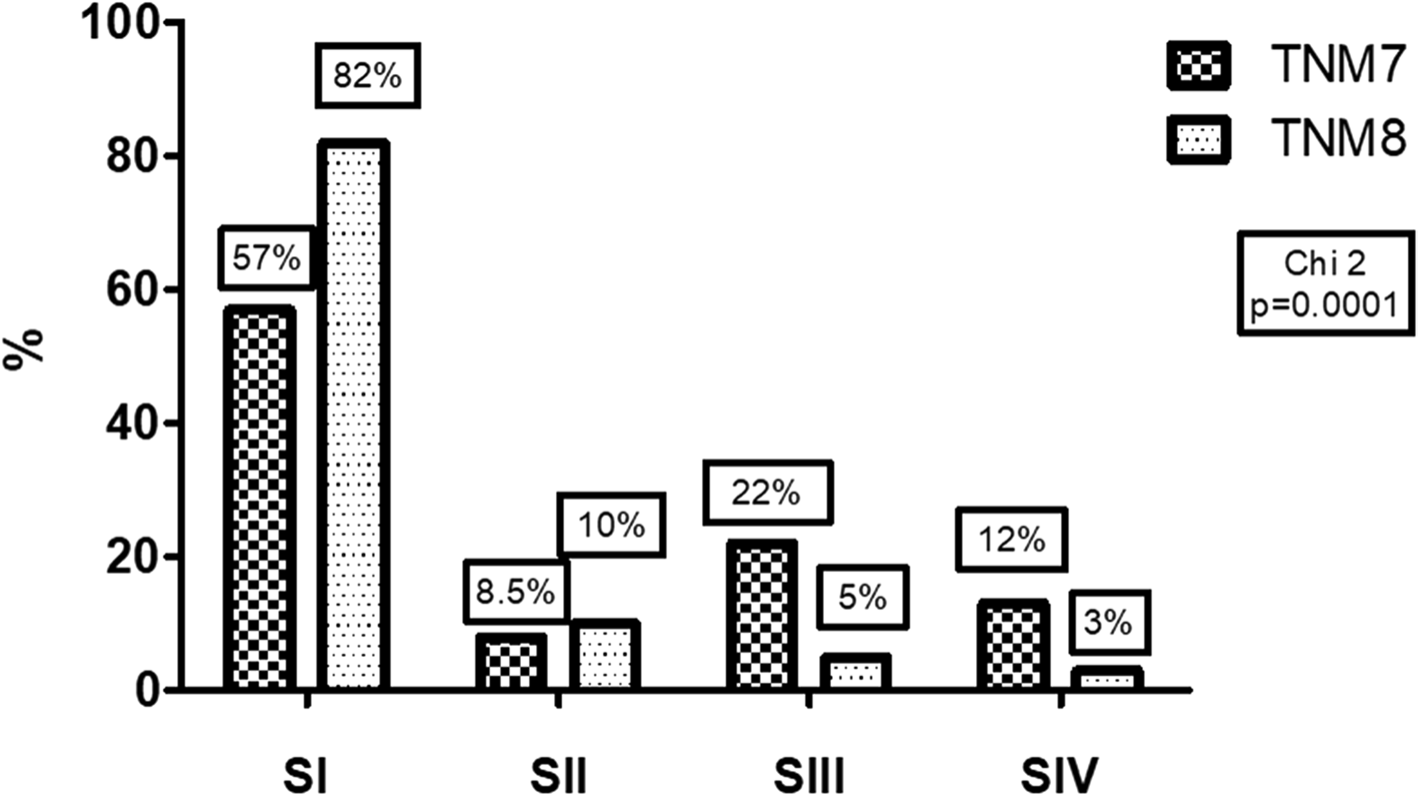
Comparison of DTC patients in each stage of TNM8 and TNM7 after reclassification

Comparison between each stage in TNM8 vs. TNM7 resulted as follows: 82% vs. 57% of patients in SI, 10% vs. 8.5% in SII, 5% vs. 22% in SIII, and 3% vs. 12% in SIV. A pattern of increase in the number of patients in stages I and II and a similar decrease in stages III and IV after reclassification was observed (Fig. 1).

### ATA recurrence risk assessment and clinical status at the end of follow-up within TNM7 and 8

The population’s characteristics regarding ATA Risk of recurrence in each TNM7 and TNM8 stages are shown in Table 3.

**Table 3:** ATA Risk of Recurrence in each stage of TNM7 and TNM8

ATA Risk	TNM7 n (%)				TNM8 n (%)			
	I	II	III	IV	I	II	III	IV
Low	103(75)	15(11)	16(12)	3(2)	130(95)	6(4)	0(0)	1(1)
Intermediate	24(32)	5(6)	37(49)	9(12)	57(76)	14(19)	3(4)	1(1)
High	12(36)	1(3)	1(3)	19(57)	16(48)	3(9)	9(27)	5(15)

Abbreviations: *TNM* tumor-node-metastasis, *TNM7* 7th edition of TNM staging system, *TNM8* 8th edition of TNM staging system, *ATA* American Thyroid Association, *S* stage

Applying TNM8, stage I patients were stratified as low risk in 130/203 (64%) cases, as intermediate risk in 57/203 (28%), and as high risk in 16/203 (8%). Those in stage II were classified as low risk in 6/23 (26%), as intermediate risk in 14/23 (61%), and as high risk in 3/23 (13%). In stage III, no patient was classified as low risk, 3/12 (25%) as intermediate risk and 9/12 (75%) as high risk, and stage IV patients were classified as low risk in 1/7 (14%), intermediate-risk 1/7 (14%) and as high risk 5/7 (71%).

The proportion of low-risk patients in SI increased significantly from 75 to 95% (*p* < 0.0001) after TNM7 to TNM8 reclassification, showing that several low-risk patients had been previously misclassified in higher risk for mortality stages. However, the proportion of patients with intermediate/ high ATA risk of recurrence in Stage I was significantly higher in TNM8 (68%) than in TNM7 (33%) (*p* < 0.0001).

The recurrence/persistence of disease was analyzed by comparing both classifications, and only in SIII, a significant increase was demonstrated: 66% TNM8 vs. 13% TNM7 (*p* = 0.0023).

In the segment of patients between 45 and 55 years old, none had distant metastases, so all were assigned to SI TNM8. Clinical status observed at the end of follow-up for those patients in TNM8 was: 66% with NED, 9.4% with structural persistence (1 death), 11% with undetermined response, 2% with biochemical persistence, and 11% of the patients were lost at the end of follow-up.

### Disease-specific mortality

There were 4 disease-specific deaths in the study group. Crude disease-specific death rate: 4/245 = 1.6%, occurred at 36, 60, 96 and 144 months of follow-up.

Out of these four patients, all SIV TNM7, 3 remained SIV in TNM8, and one patient aged less than 55 years was downstaged to SI TNM8.

**Fig. 2:**
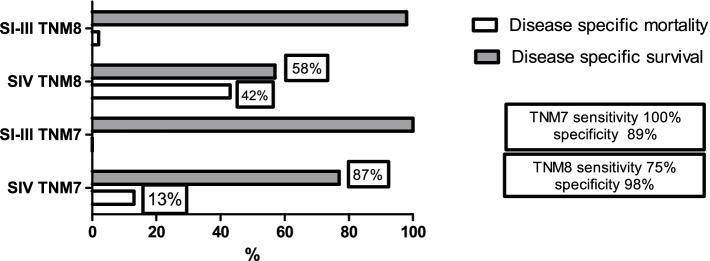
Comparison of Disease-Specific Mortality in SI-III vs. SIV between TNM7 and TNM8

Regarding SIV TNM7 vs. TNM8, a trend for higher mortality in TNM8 was observed: 13% TNM7 vs. 42% TNM8 (*p* = 0.1). Sensitivity and specificity in SIV TNM 7 were 100 and 89%, respectively, and in SIV TNM8, 75 and 98%, respectively (Fig. 2). A higher likelihood ratio for mortality was observed in those patients stratified SIV TNM8 vs. SIV TNM7 (LR 45.1 vs. LR 8.9). In contrast, in SII and SIII, no differences between both editions were attained since no patient died due to a specific cause of disease.

Applying Kaplan-Meier analysis with censored patients considered, the Disease-Specific Survival (DSS) rate was estimated. For the whole study group, the 10-year DSS rate was 96.9% (95% CI: 99.4–99.6%). In TNM7, the 10-year DSS rates for SI, SII, SIII and SIV were 100, 100, 100% y 74%, respectively. In TNM8 the same rates were 97.6, 100, 100 and 37.5%, respectively.

**Fig. 3:**
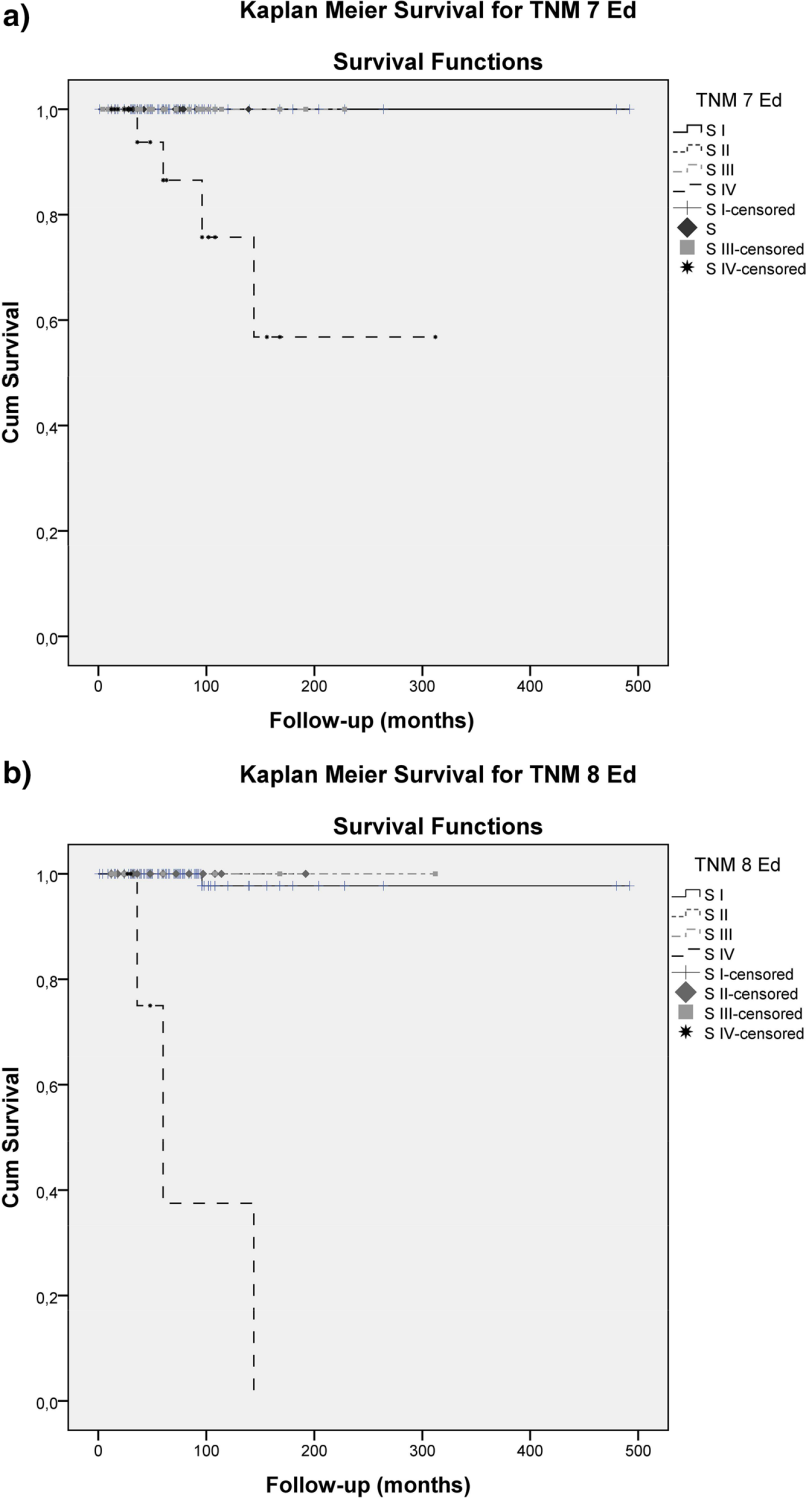
Kaplan Meier cancer-specific survival curves according to the (**a**) TNM7 and (**b**) TNM8 staging system

Disease-specific mortality (DSM) was significantly different according to TNM7 and TNM8, as shown in Fig. 3 (*P* < 0.001 and *P* < 0.001 of the log-rank test, respectively).

Patients in stage IV TNM8 had a significantly poorer DSS than those with stage I. The HR of stage IV was 97.8 (95% CI 9.6–995) (*p* < 0.001) while, within TNM 7, stage IV did not attain statistically significant difference in terms of survival with regards to stage I (*p* = 0.6 by the Cox Proportional Hazard model).

The proportion of variance explained (PVE), expressed as R-Squared (%) for TNM8 was = 0.309 (Adjusted R-Squared = 0.299) (29%) and for TNM 7, R-Squared was 0.143 (Adjusted R-Squared = 0.130) (13%). The evidence suggests that PVE for TNM8 was more than twice that of TNM7.

## Discussion

In this study, we have validated the impact of the new TNM8 edition classification in two tertiary hospitals in Argentina in terms of mortality prognosis and clinical status at the end of follow-up. After applying TNM8 and downstaging 40% of the population, a higher trend of mortality in SIV and a more significant proportion of disease persistence in SIII were revealed compared to the older edition. Our results are consistent with previously published studies [8, 9, 14, 15] and generate an antecedent for Latin America, where information about this topic is still scarce.

An essential modification in the newer version TNM8 has been the definition of a different age cut-off. Since the former and second edition of TNM was published in 1983, the cut-off age of 45 years has been used as a dichotomous variable in patients with DTC. Age has always been an important prognostic factor and it has been incorporated into different staging systems used in clinical practice [16, 17].

A growing number of studies indicate that the age cut-off of 45 years may not be statistically as robust as established in the TNM7 classification system. It can lead to over staging of many low-risk patients [18]. In the multi-institutional study published by Nixon et al. [5] with a cohort of 9484 patients, TNM7 was used. When the cut-off point of 55 years was employed, 12% of the entire cohort was understaged, and 10-year disease-specific survival of 98% was observed in this particular set of patients.

Moreover, in a cohort of 6333 patients from two centers in Seoul, Korea, recruited between 1995 and 2005, different age cutoff points between 50 and 65 years with 5-year intervals were tested. It was observed that mortality increased from 55 years onwards, and by using this age cut-off, 20% of patients could be adequately downstaged [18].

Another study compared mortality and clinical disease status between DTC patients < 55 and = 55 years. It was observed that age was a predictor of mortality in patients with a high risk of recurrence and structural persistence of disease, with better outcomes in terms of survival in patients < 55 than ≥55 years, 74% vs. 12%, (*p* = 0.001) respectively [19]. More recently, Trimboli et al. [20] described DTC patients older than 55 years with significantly higher risk of relapse over time only in the high ATA recurrence risk group and Van Velsen et al. [21] when retrospectively studied adult patients with ATA high-risk DTC, also, identified an age cut-off of 50 or 60 years for PTC and of 65 or 70 years for FTC as a significant negative influence on disease outcomes.

It is of particular interest to analyze the results of the Surveillance, Epidemiology, and End Results (SEER) database with 31,802 participants. It was demonstrated that 10-years DSS was inversely associated with an increase in age; however, the association between age and cancer-specific mortality was linear, without a clear cut-off point of age [22].

In the present study, mortality was explored in each stage of TNM, and it was revealed that stage IV of TNM8 was more specific for DSM than TNM7. Moreover, the PVE was doubled by TNM8. These findings suggest that the new classification allows for a better prediction of DSS by reducing the number of patients with a fair clinical outcome from SIII and IV.

Many downstaged patients reflect the advantage of TNM8 to lower risk categories requiring less complex therapeutic strategies. In this study, almost half of the population was downstaged, in accordance with other studies published in the literature. As observed by Kim TH et al. [8], in their cohort of 3176 Korean patients with DTC between 1996 and 2005, 37.6% of patients were downstaged using TNM8.

In line with these reports, Kim M et al. [9] also described 38% of downstaging in a cohort of 1613 DTC patients. Other authors also concluded that aggressive treatments could be safely avoided in the long-term follow-up when patients are stratified as having a lower risk of mortality [11].

To further improve the prognostic accuracy of TNM8, Ito et al. [23] subdivided tumor extension (T4a) into T4a1 and T4a2 based on intraoperative gross findings and N1 according to size (< 3 cm and ≥ 3 cm) based on preoperative imaging findings. Therefore, a subdivision of clinical tumor extension and node metastasis can also be considered of clinical relevance to identify poor-risk patients more accurately.

Although the TNM classification was created to evaluate mortality in patients with DTC, it is worth noting that in the present study, a significantly higher proportion of patients with structural persistence was observed in SIII TNM8 compared to TNM7, as also demonstrated by Thai research [12] These findings can be explained by the fact that all tumors with gross local extension were eventually downstaged from SIV.

In spite of the fact that the ATA classification predicts recurrence outcomes and AJCC/TNM predicts survival, a few studies have combined both systems, reaching better predictions with both classifications. Regarding initial ATA risk of recurrence classification, it was shown that TNM8 allowed for a better distribution of patients classified in SI and SII within the categories of low/intermediate risk of recurrence. In contrast, those patients with SIII and SIV were better discriminated within the ATA high-risk category than TNM7. Similarly, Nava et al. [14] showed that with TNM8, patients with low risk of recurrence were primarily located in SI and SII, and those with a high risk of recurrence in SIII and SIV, while in TNM7, the groups were not as well discriminated, suggesting that TNM8 is a good predictor of relapses and complications from the disease.

Consequently, if the risk of recurrence together with age was considered within the TNM classification, a more individualized survival prognostic could be attained, especially in patients with a high risk of recurrence, instead of a “one size fits all” classification system [24]. Indeed, Kim YN et al. have proposed incorporating multiple age cut-off points and essential histological information to facilitate the estimation of survival and avoid underestimating the effect of age as it increases [25].

It is also worth noting that with the new definition of T3 classification, a large proportion of patients were shifted from SIII and IV to SII. Consequently, patients with lymph node metastases and extra-thyroidal extension were also downstaged compared to the previous edition. Therefore, although TNM8 has good accuracy in establishing mortality, these patients should not be underestimated about the risk of recurrence, especially in SII [13].

One of the limitations of the present study is its retrospective design. However, this is a typical pattern of most observational studies on DTC in whom long-term follow-up of patients is required. The relatively small number of patients, despite having gathered data from 2 centers, has also precluded better-estimating mortality rates.

In conclusion, we have confirmed in an Argentinian cohort of patients the higher diagnostic accuracy of TNM8 compared to TNM 7. Many patients were downstaged who would have been otherwise unnecessarily classified as high risk for mortality and followed more intensively. Although stage IV became more specific for mortality prediction, those patients between 45 and 55 years with locally advanced disease assigned to SI should still receive special care.

These findings contribute to developing future Latin American Guidelines on thyroid cancer based on local experience.

## Data Availability

The datasets used and analyzed during this study are available from the corresponding author on reasonable request.

## References

[1] Tuttle M, Morris LF, Haugen B, Shah J, Sosa JA, Rohren E, Amin MB, Edge SB, Greene F, Byrd D, Brookland RK, Washington MK, Gershenwald JE, Compton CC, Hess KR, Sullivan DC, Jessup JM, Brierley J, Gaspar LE, Schilsky RL, Balch CM, Winchester DP, Asare EA, Madera M, Gress DM, Meyer LR (2017). Thyroid-differentiated and anaplastic carcinoma (chapter 73). AJCC Cancer staging manual.

[2] Haugen BR, Alexander EK, Bible KC, Doherty GM, Mandel SJ, Nikiforov YE (2016). 2015 American Thyroid Association management guidelines for adult patients with thyroid nodules and differentiated thyroid cancer. Thyroid.

[3] Van Velsen E, Stegenga MT, van Kemenade FJ, Kam BL, van Ginhoven TM, Visser WE (2018). Comparing the prognostic value of the eighth edition of the AJCC/TNM staging system between papillary and follicular thyroid cancer. Thyroid.

[4] Ito Y, Miyauchi A, Hirokawa M, Yamamoto M, Oda H, Masuoka H (2018). Prognostic value of the 8th edition of the tumor-node -metastasis classification for patients with papillary thyroid carcinoma: a single-institution study at a high volume center in Japan. Endocr J.

[5] Nixon IJ, Wang L, Migliacci J, Eskander A, Campbell M, Aniss A (2016). An international multi-institutional validation of age 55 years as a cut-off for risk stratification in the AJCC/UICC staging system for well differentiated thyroid Cancer. Thyroid.

[6] Ito Y, Tomoda C, Uruno T, Takamura Y, Miya A, Kobayashi K (2006). Prognostic significance of extrathyroid extension of papillary thyroid carcinoma: massive but not minimal extension affects the relapse-free survival. World J Surg.

[7] Woo CG, Sung CO, Choi YM, Kim WG, Kim TY, Shong YK (2015). Clinicopathological significance of minimal Extrathyroid extension in solitary papillary thyroid carcinomas. Ann Surg Oncol.

[8] Kim TH, Kim YN, Kim HI, Park SI, Choe JH, Kim JH (2017). Prognostic value of the eighth edition AJCC TNM classification for differentiated thyroid carcinoma. Oral Oncol.

[9] Kim M, Kim WG, Oh HS, Park S, Kwon H, Song DE, Kim TY (2017). Comparison of the 7th and 8th editions of the AJCC/UICC TNM staging system for differentiated thyroid Cancer. Thyroid.

[10] Tam S, Boonsripitayanon M, Amit M, Fellman BM, Li Y, Busaidy NL (2018). Survival in differentiated thyroid Cancer: comparing the AJCC Cancer staging seventh and eighth editions. Thyroid.

[11] Pontius LN, Oyekunle TO, Thomas SM, Stang MT, Scheri RP, Roman SA (2017). Projecting survival in papillary thyroid Cancer: a comparison of the 7th and 8th editions of the AJCC/UICC staging Systems in two Contemporary National Patient Cohorts. Thyroid.

[12] Thewjitcharoen Y, Chatchomchuan W, Karndumri K, Porramatikul S, Krittiyawong S, Wanothayaroj E (2021). Impacts of the American joint committee on Cancer (AJCC) 8 th edition tumor, node, metastasis (TNM) staging system on outcomes of differentiated thyroid cancer in Thai patients. Heliyon.

[13] Shteinshnaider M, Kalmovich LM, Koren S, Or K, Cantrell D, Benbassat C (2018). Reassessment of differentiated thyroid Cancer patients using eighth TNM/AJCC classification system: a comparative study. Thyroid.

[14] Nava CF, Zanella AB, Scheffel RS, Maia AL, Dora JM (2019). Impact of the updated TNM staging criteria on prediction of persistent disease in a differentiated thyroid carcinoma cohort. Arch Endocrinol Metab.

[15] Godoi Cavalheiro B, Luongo de Matos L, Kober Nogueira Leite A, Vamondes Kulcsar MA, Cernea CR, Kowalski LP (2021). Survival in differentiated thyroid carcinoma: Comparison between the 7th and 8th editions of the AJCC/UICC TNM staging system and the ATA initial risk stratification system. Head Neck.

[16] Edge SB, Compton CC (2010). The American joint committee on Cancer: the 7th edition of the AJCC cancer staging manual and the future of TNM. Ann Surg Oncol.

[17] Kim TY, Kim WG, Kim WB, Shong YK (2014). Current status and future perspectives in differentiated thyroid cancer. Endocrinol Metab.

[18] Kim M, Kim YN, Kim WG, Park S, Kwon H, Jeon MJ (2016). Optimal cutoff age in the TNM staging system of differentiated thyroid Cancer: is 55 years better than 45 years?. Clin Endocrinol Ox.

[19] Shah S, Boucai L (2018). Effect of age on response to therapy and mortality in patients with thyroid Cancer at high risk of recurrence. J Clin Endocrinol Metab.

[20] Trimboli P, Piccardo A, Signore A, Valabrega S, Barnabei A, Santolamazza G (2020). Patient age is an independent risk factor of relapse of differentiated thyroid carcinoma and improves the performance of the ATA stratification system. Thyroid.

[21] Van Velsen EFS, Peeters RP, Stegenga MT, Van Kemenade FJ, Van Ginhoven TM, Verburg FA (2021). The influence of age on disease outcome in 2015 ATA high-risk differentiated thyroid cancer patients. Eur J Endocrinol.

[22] Adam MA, Thomas S, Hyslop T, Scheri RP, Romas SA, Sosa JA (2016). Exploring the relationship between patient age and Cancer-specific survival in papillary thyroid Cancer: rethinking current staging systems. J Clin Oncol.

[23] Ito Y, Miyauchi A, Kihara M, Masuoka H (2020). HigashiyamaT, Miya a. subclassification of tumor extension and nodal metastasis in papillary thyroid Cancer to improve prognostic accuracy of the eighth edition of the tumor-node-metastasis classification. World J Surg.

[24] Ghaznavi SA, Ganly I, Shaha AR, English C, Wills J, Tuttle RM (2018). The ATA risk stratification system is used to refine and individualize the AJCC 8th edition disease-specific survival estimates in differentiated thyroid cancer. Thyroid.

[25] Kim YN, Kim M, Ahn HS, Kim K, Park SY, Kim HI (2019). Refining the tumor-node-metastasis staging system for individualized treatment of differentiated thyroid carcinoma. Oral Oncol.

